# Follow-Up Period Affects the Association between Serum 25-Hydroxyvitamin D Concentration and Incidence of Dementia, Alzheimer’s Disease, and Cognitive Impairment

**DOI:** 10.3390/nu16183211

**Published:** 2024-09-23

**Authors:** William B. Grant

**Affiliations:** Sunlight, Nutrition, and Health Research Center, 1745 Pacific Ave., Suite 504, San Francisco, CA 94109, USA; wbgrant@infionline.net

**Keywords:** Alzheimer’s disease, cognitive impairment, dementia, follow-up period, neurological conditions, risk, vitamin D deficiency

## Abstract

Background/Objectives: Vitamin D’s effect on risk health outcomes is often evaluated using prospective cohort studies. For vitamin D, risk ratios (RRs) are based on health outcomes with respect to serum 25-hydroxyvitamin D [25(OH)D] concentrations measured at time of enrollment. Serum 25(OH)D concentrations vary over time, thereby diluting the effect of 25(OH)D for long follow-up periods. Inverse relationships between RR and follow-up period have been reported for all-cause mortality rate and cancer incidence rates. Here, the effect for neurological outcomes is evaluated. Methods: The analysis examines how follow-up period affected results from nine cohort studies of all-cause dementia, six studies of Alzheimer’s disease, and nine for cognitive impairment with respect to vitamin D deficiency. Results: For all-cause dementia, Alzheimer’s disease, and cognitive impairment, respectively, the linear regression fits are RR = 2.9 − 0.14 × years, *r* = 0.73, *p* = 0.02; RR = 2.9 − 0.14 × years, *r* = 0.69, *p* = 0.13; and RR = 1.8 − 0.066 × years, *r* = 0.72, *p* = 0.03. The regression fit to RR for the shortest follow-up period for each outcome is considered the best estimate of vitamin D deficiency’s effect on risk. Those values are approximately twice that found by averaging all RRs without considering the effect of follow-up period. Conclusions: Vitamin D’s effect on risk of neurological conditions is inversely correlated with mean follow-up period in prospective cohort studies. This effect should be considered in the design and analysis of such studies. Additional studies should also be conducted regarding raising serum 25(OH)D concentrations to reduce risk of brain function decline.

## 1. Introduction

Prospective cohort studies are often used to ascertain how lifestyle, diet, nutrients, lifestyle, and biomarkers are related to health outcomes. The standard procedure is to enroll participants, obtain values for factors to be studied and those that might affect the outcome, monitor participants for several years, and note changes in the health condition of interest. Because serum 25-hydroxyvitamin D [25(OH)D] concentrations change for various reasons, relying only on the 25(OH)D concentration measured at the time of enrollment is problematic.

Since at least 1999, researchers have known that long-term follow up in prospective studies results in ”regression dilution” [1]. Of the articles consulted in preparing this article, only Kuzma and colleagues (2016) [2] cited that article. Since 2011, that same effect has been found in prospective cohort studies regarding serum 25(OH)D and cancer [3] and, since 2012, all-cause mortality rate [4]. An observational study in Norway reported that “depending on the method of adjusting for season, the correlation coefficient between serum 25(OH)D measurements from 1994 and 2008 ranged from 0.42 to 0.52” [5]. A more recent report showed that the analysis of the risk of colorectal cancer with respect to serum 25(OH)D concentration on the basis of prospective cohort studies [6] was incorrect because the researchers had not realized that men had nearly four times the rate of change in relative risk (RR) with respect to follow-up time as women (Figure 1 in [7]).

The article by Zhang and colleagues [8] regarding the association between vitamin D levels and risk of dementia, Alzheimer’s disease (AD), and cognitive impairment forms the basis for the present article. The authors used the standard random effects model regarding 17 prospective cohort studies with 486,921 individuals. For dementia with respect to vitamin D deficiency (VDD), RR = 1.42 (95% confidence interval [CI], 1.21–1.65). However, that analysis did not consider the effect of each study’s mean follow-up period. The present study plots the RR values for the various health outcomes vs. mean follow-up period. The plots show that RR is highest for the shortest follow-up periods and declines to near 1.00 for follow-up periods near 13 years. This study examines the implications of follow-up period in prospective cohort studies on the estimation of the effect of VDD on risk of the three adverse brain health outcomes. The analysis suggests that using the RR for the prospective studies with the shortest follow-up period results in an estimate of the effect of VDD being about twice as high as from an analysis that ignores the length of follow-up period.

## 2. Materials and Methods

The sources of the data used in this study were obtained from two meta-analyses [8,9] as well as a search of Google Scholar for additional studies not included in those two meta-analyses. All of the studies in the meta-analyses were added to the data tables in this study. However, studies that did not provide follow-up period, the comparison of serum 25(OH)D concentration between participants with or without adverse brain health, or were based on dietary vitamin D intake were not included in the analyses. One study, (Graf, 2014) [10], was omitted from the dementia analysis due having a very large range of 95% CI.

To evaluate how follow-up period affects risk of dementia with respect to VDD, several sources were used. Much of the data are from Figure 2 from Zhang and colleagues [8] plus the results from Figure 2 in Chen and colleagues [9]. Data for AD were obtained from Figure 3a in Zhang and colleagues [8]. Data for cognitive impairment (CogImp) are from Figure 3b in Zhang and colleagues [8]. Years of follow-up were obtained from Chen and colleagues [9] or from the original studies. Table 1, Table 2, Table 3, Table 4 and Table 5 show the relevant information regarding the data in the cohort studies. For dementia, mean ages of participants at baseline ranged from 53 (SD 17) to 85 (SD 7) years. The mean 25(OH)D concentrations for studies that gave values ranged from 32 (standard deviation [SD] 25) to 69 ± 19 nmol/L. The 25(OH)D comparisons included <25 versus >50 mol/L, <50 versus >50 mol/L, <50 versus ≥75 nmol/L, and so on. The mean follow-up period ranged from 5.6 to 30 years. For CogImp, mean ages of participants at baseline ranged from 67 ± 5 to 74 (SD 7) years. Mean 25(OH)D concentrations for studies that gave values ranged from 50 (SD 21) to 84 (SD 54) nmol/L. The 25(OH)D comparisons included <25 versus ≥50 mol/L, <50 versus ≥75 nmol/L, and so on. The mean follow-up period ranged from 4.0 to 13 years.

In the analysis, it is assumed that the only important factor is the mean follow-up period. Though values for various factors could affect the HR, in the analysis it appears that they are smaller than the effect of follow-up period. Studies with mean follow-up period greater than 15 years were omitted because those periods were considered too long to yield meaningful data. One study (Graf, 2014 [10] was omitted from the dementia analysis due to having very large 95% CI range due to the low numbers of participants and those who developed dementia.

**Table 1:** Data for vitamin D deficiency and risk of dementia or Alzheimer’s disease from Figure 2 and Figure 3a in Zhang and colleagues (2024) [8] plus a recent study from the UK Biobank [11].

Country	Mean Age (±SD) (yrs)	*N*_T_	*N*_D_	*N*_AD_	Author, yr, Ref.
USA	74 ± 5	1658	171	102	(Littlejohns, 2014) [12]
Germany	84 ± 3	861 F, 473 M	250	209	(van Lent, 2022) [13]
Israel	53 ± 17	2454 F, 1824 M	133		Kiderman, 2023) [14]
UK	64.6	13,486	283	101	(Geng, 2022) [15]
USA	72 ± 7	1663	267	208	(Karakis, 2016) [16]
Norway	78	790 F, 644 M	324		(Asante, 2023) [17]
France	73 ± 5	916	177	124	(Féart, 2017) [18]
Sweden	71	1182 M	250 M	116 M	(Olsson, 2017) [19]
The Netherlands	69 ± 8	3462 F, 2625 M	795	641	(Licher, 2017) [20]
UK	62 ± 3	140,857 F, 128,372 M	7087	3616	(Chen, 2024) [11]
Omitted					
Switzerland	85 ± 7	147 F, 53 M	46		(Graf, 2014) [10]
USA	62	793 B, 859 W	145		(Schneider, 2014) [21]
Finland	Cases: 69 ± 7 Noncases: 56 ± 10	2724 F, 2286 M	100 F, 51 M		(Knekt, 2014) [22]
USA	57 ± 6	13,039	1323		(Fashanu, 2019) [23]
Denmark	58	10,186	418	92	(Afzal, 2014) [24]

Key: B, black; F, female; M, male; *N*_AD_, number developing Alzheimer’s disease; *N*_D_, number with dementia; *N*_T_, total; SD, standard deviation; W, white.

**Table 2:** Data for vitamin D deficiency and risk of dementia from Figure 2 in Chen and colleagues [9] and Figure 2 in Zhang and colleagues (2024) [8].

Mean BMI (±SD) (kg/m^2^)	Mean 25(OH)D (±SD) (nmol/L)	25(OH)D Comparison (nmol/L)	Mean Follow-Up (yrs)	RR (95% CI)	Author, yr, Ref.
27 ± 5		<25 vs. >50	5.6	2.18 (1.18–4.02)	(Littlejohns, 2014) [12]
27 ± 6	54 ± 24	<25 vs. >50	7	2.38 (1.31–4.23)	Kiderman, 2023) [14]
27 ± 5	63 ± 28		9	1.00 (0.58–1.72)	(Karakis, 2016) [16]
27 ± 3	50 ± 21	<50 vs. >50	10	1.09 (0.64–1.83)	(Asante, 2023) [17]
26 ± 4		<50 vs. >50	11.4	2.12 (1.21–3.71)	(Féart, 2017) [18]
26 ± 3	69 ± 19	<50 vs. ≥75	12	0.86 (0.58–1.30)	(Olsson, 2017) [19]
27 ± 4	49 (IQR 30–69)	<25 vs. >50	13.3	1.22 (0.98–1.54)	(Licher, 2017) [20]
27 ± 4	50 ± 21	<50 vs. >50	13.6	1.25 (1.16–1.34)	(Chen, 2024) [11]
Omitted	from analysis due to long follow-up period				
23 ± 4	32 ± 25	<25 vs. >75	2	2.85 (0.45–17.95)	(Graf, 2014) [10]
27 ± 5, W	64 ± 20 W;	High vs. low tertile	16.6	1.30 (0.62–2.71)	(Schneider, 2014) [21]
30 ± 6, B	43 ± 16 B	High vs. low tertile	16.6	1.81 (0.33–6.50)	(Schneider, 2014) [21]
26 ± 4 F	Cases: 40 ± 20 Noncases: 43 ± 17	High vs. low quartile	17	3.03 (1.37–6.69)	(Knekt, 2014) [22]
26 ± 4 M	Cases: 40 ± 20 Noncases: 43 ± 17	High vs. low quartile	17	1.35 (0.53–3.44)	(Knekt, 2014) [22]
28 ± 5	61 ± 22	<25 vs. >50	20	1.24 (1.05–1.48)	(Fashanu, 2019) [23]
25 ± 3	45 (M) 40 (F)	<25th vs. >50th percentile	30	1.27 (1.01–1.60)	(Afzal, 2014) [24]

Key: 25(OH)D, 25-hydroxyvitamin D; 95% CI, 95% confidence interval; B, black; BMI, body mass index; F, female; IQR, interquartile range; M, male; RR, relative risk; SD, standard deviation; W, white.

**Table 3:** Data for vitamin D deficiency and risk of Alzheimer’s disease from Figure 2 in Chen and colleagues [9] and Figure 3a in Zhang and colleagues (2024) [8].

Mean BMI (±SD) (kg/m^2^)	Mean 25(OH)D (±SD) (nmol/L)	25(OH)D Comparison (nmol/L)	Mean Follow-Up (yrs)	RR (95% CI)	Author, yr, Ref.
27 ± 5		<25 vs. >50	5.6	2.20 (1.01–4.80)	(Littlejohns, 2014) [12]
26 ± 4	37 (IQR 25–58)	<25 vs. >50	7	2.28 (1.47–3.53)	(van Lent, 2022) [13]
31 ± 5		<25 vs. >50	8.5	1.72 (1.02–2.91)	(Geng, 2022) [15]
27 ± 5	63 ± 28		9	0.72 (0.37–1.42)	(Karakis, 2016) [16]
26 ± 4		<50 vs. >50	11.4	2.85 (1.36–5.97)	(Féart, 2017) [18]
26 ± 3	69 ± 19	<50 vs. ≥75	12	1.19 (0.67–2.12)	(Olsson, 2017) [19]
27 ± 4	50 ± 21	<50 vs. >50	13.6	1.19 (1.07–1.31)	(Chen, 2024) [11]
Omitted	from analysis due to long follow-up period				

Key: 25(OH)D, 25-hydroxyvitamin D; 95% CI, 95% confidence interval; BMI, body mass index; RR, relative risk; SD, standard deviation.

**Table 4:** Data for vitamin D deficiency and risk of cognitive impairment from Figure 3b in Zhang and colleagues (2024) [8].

Country	Mean Age (±SD) (yrs)	*N*	*N*_CI_	Test	Author, yr, Ref.
USA	74 ± 5	1812 F	446, 409	MMSE, TMTB	(Slinin, 2012) [25]
Italy	74 ± 7	1208 F, 719 M	466	MMSE	(Toffanello, 2014) [26]
USA	74 ± 6	806 M	126	MMSE, TMTB	(Slinin, 2010) [27]
Italy	74 ± 7	487 F, 370 M		MMSE	(Llewellyn, 2011) [28]
USA	72 ± 3	1750 F, 832 M	324	BVRT	(Kuzma, 2016 [CHS]) [2]
Chile	67 ± 5	666 F, 289 M	54	MMSE	(Marquez, 2022) [29]
Norway	78	790 F, 644 M	717	MoCA	(Asante, 2023) [17]
Sweden	71	1182 M	80	MMSE	(Olsson, 2017) [19]
The Netherlands	74 ± 6	1010 F, 820 M	346	RAVLT	(Kuzma, 2016 [LASA]) [2]

Key: BVRT, Benton Visual Retention Test; CHS, Cardiovascular Health Study; F, female; LASA, Longitudinal Aging Study Amsterdam; M, male; MMSE, Mini-Mental State Examination; MoCA, Montreal Cognitive Assessment; *N*, number of participants; *N*_CI_, number with cognitive impairment; RAVLT, Rey’s Auditory Verbal Learning Test; SD, standard deviation; TMTB, Trail Making Test Part B.

**Table 5:** Data for vitamin D deficiency and risk of cognitive impairment from Figure 3b in Zhang and colleagues (2024) [8].

Assessment (yrs)	Mean BMI (±SD) (kg/m^2^)	Mean 25(OH)D (±SD) (nmol/L)	25(OH)D Comparison (nmol/L)	Mean Follow-Up (yrs)	RR (95% CI)	Author, yr, Ref.
2 and 4	26 ± 5, F		<25 vs. ≥75	4.0	1.45 (1.10–1.86)	(Slinin, 2012) [25]
4	27 ± 3	84 ± 54	<50 vs. ≥75	4.4	1.36 (1.04–1.80)	(Toffanello, 2014) [26]
4.6	27 ± 3, M		<50 vs. ≥75	4.6	1.29 (0.91–1.74)	(Slinin, 2010) [27]
3 and 6		52 ± 37	<25 vs. ≥75	5.2	1.64 (1.20–2.05)	(Llewellyn, 2011) [28]
Annual	27 ± 5		<25 vs. ≥50	6.5	1.73 (1.22–2.45)	(Kuzma, 2016 [CHS]) [2]
?	29 ± 5, F 28 ± 4, M	Cases: 58 ± 32 Noncases: 71 ± 38	30–48 vs. >75	9.6	1.25 (0.64–2.85)	(Marquez, 2022) [29]
	27 ± 3	50 ± 21	<50 vs. >50	10	1.06 (0.73–1.44)	(Asante, 2023) [17]
	26 ± 3	69 ± 19	<50 vs. ≥75	12	0.67 (0.31–1.36)	(Olsson, 2017) [19]
Every 3–4	27 ± 4		<25 vs. ≥50	13	1.12 (0.84–1.48)	(Kuzma, 2016 [LASA]) [2]

Key: 25(OH)D, 25-hydroxyvitamin D; 95% CI, 95% confidence interval; BMI, body mass index; F, females; HR, hazard ratio; M, males; RR, relative risk; SD, standard deviation.

Table 4 and Table 5 give the data associated with the CI studies. The numbers of cognitively normal participants at baseline and the number who developed CI are for those in the 25(OH)D categories used in the HR or OR analyses.

These data were used to examine the effect of follow-up period in the risk of dementia, AD, and CogImp as shown in the results section.

## 3. Results

**Figure 1:**
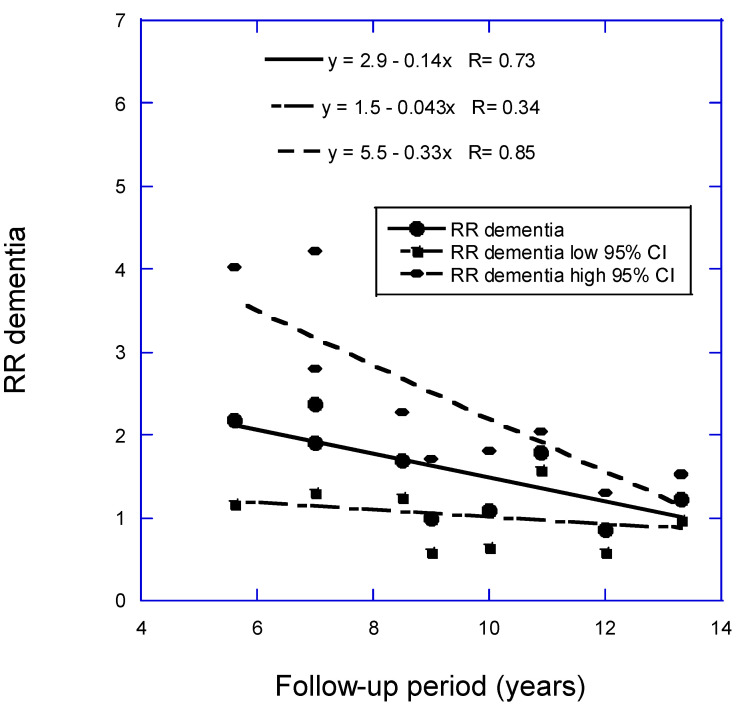
Scatter plot of relative risk (RR) versus low to high 25(OH)D concentration for dementia with respect to mean follow-up period less than 15 years from Figure 2 in Zhang and colleagues (2024) [8] plus Chen and colleagues (2024) [11] but omitting Féart and colleagues (2017) [18]. Key: 95% CI, 95% confidence interval.

In the analysis for dementia, omitted were one study with high uncertainty, accounting for only 0.7% of the weight, and three studies with follow-up periods of 17+ years. Two studies were conducted, one with 11 studies and one with 10, omitting Féart and colleagues [18]. The linear fit to the data with 11 studies is RR = 2.8 − 0.12 × years, *r* = 0.59, *p* = 0.03. The linear fit to the data with 10 studies is RR = 2.9 − 0.14 × years, *r* = 0.73, *p* = 0.02 (Figure 1). If the Graf study [10] is included, the regression fit is RR = 3.0 − 0.16 × years, *r* = 0.84, *p* = 0.001. (For 13.3 years, two studies reported RR = 1.22.) Chen and colleagues [9] calculated a pooled RR = 1.39 (95% CI, 1.14–1.47) for low vs. high 25(OH)D concentration based on data from 12 prospective cohort studies. Zhang and colleagues (2024) calculated an estimated pooled RR of 1.42 (95% CI, 1.21–1.65) for low vs. high 25(OH)D concentration based on data from 17 prospective cohort studies. For the shortest follow-up period, 5.6 years, the RR for the analysis with 10 studies is 2.1 (95% CI, 1.04–3.9), 2.6 times higher than the value from Zhang and colleagues, though with much larger 95% CIs.

**Figure 2:**
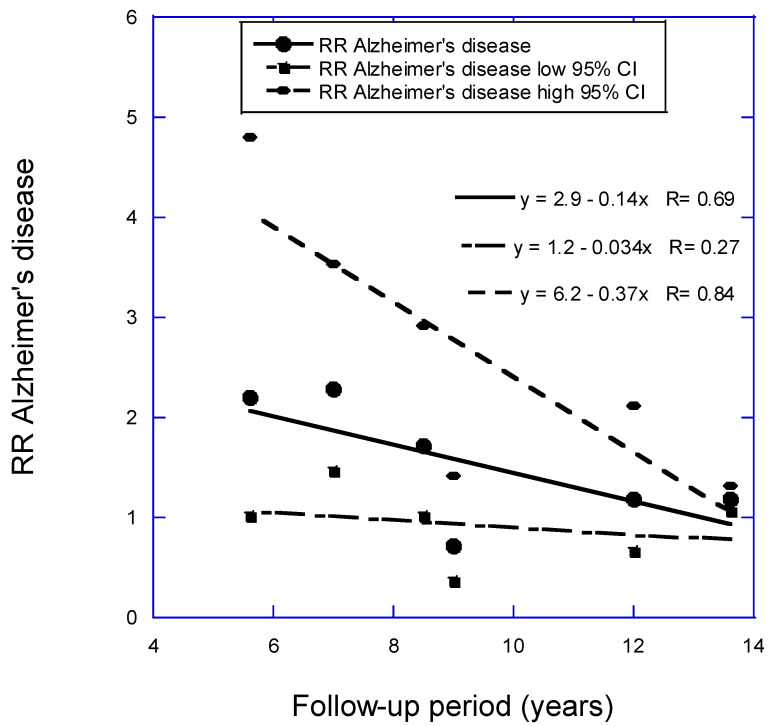
Relative risk (RR) for AD versus low to high 25(OH)D concentration versus mean follow-up period from Figure 3a in Zhang and colleagues (2024) [8] plus Chen and colleagues (2024) [11] but omitting Féart and colleagues (2017) [18]. Key: 95% CI, 95% confidence interval.

For the RR of AD versus 25(OH)D concentration as a function of follow-up period, two analyses were conducted. In the analysis with seven studies with less than 15 years of mean follow-up period in Zhang and colleagues (2024) [8] plus Chen and colleagues (2024) [11], the regression fit to the data was RR = 2.5 − 0.08 × years, *r* = 0.32, *p* = 0.48. With Féart and colleagues [18] omitted, the regression fit to the data was RR = 2.9 − 0.14 × years, *r* = 0.69, *p* = 0.13 (Figure 2). The estimated pooled RR in Zhang and colleagues [8] is 1.57 (95% CI, 1.15–2.14). The value in this article for the six studies for the shortest follow-up period, 5.6 years, is 2.12 (95% CI, 1.01–4.13). That estimate is 2.0 times higher than the estimate from Zhang and colleagues but again with higher 95% CI values.

**Figure 3:**
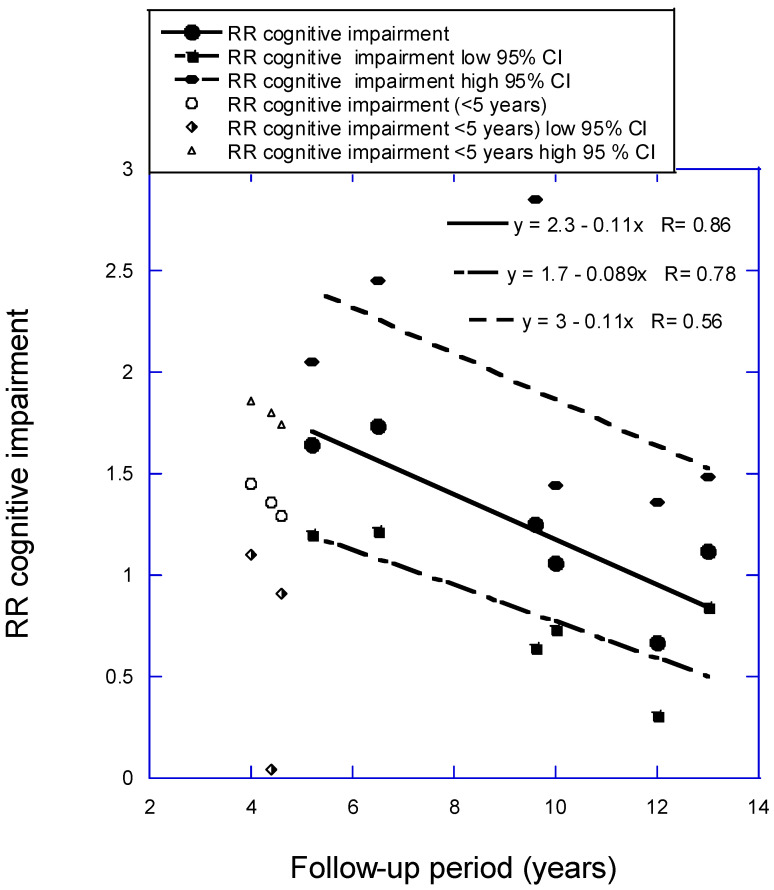
Relative risk (RR) for cognitive impairment versus low to high 25(OH)D concentration with regression fit to follow-up period for six studies with mean follow-up periods from 5 to 13 years from Figure 3b in Zhang and colleagues (2024) [8]. Key: 95% CI, 95% confidence interval; RR, risk ratio.

The analysis for CI versus 25(OH)D concentration as a function of follow-up period used six of the 10 studies in Figure 3b from Zhang and colleagues (2024) [8], with one study omitted that had very large 95% CI values and three with follow-up times less than 5 years. The regression fit to the data is RR = 2.3 − 0.11 × years, *r* = 0.88, *p* = 0.02. (If three studies with mean follow-up period between 4.0 and 4.6 years are added, RR = 1.8 − 0.066 × years, *r* = 0.72, *p* = 0.03). Figure 3 is a scatter plot of the data used in the analysis. The estimated pooled RR in Zhang and colleagues (2024) [8] for data from seven prospective cohort studies is 1.34 (95% CI, 1.19–1.52). The estimated pooled RR for AD for low vs. high 25(OH)D concentration from six prospective cohort studies in Chen and colleagues (2018) [9] is 1.28 (95% CI, 1.00–1.67). The value in this article for the six studies for the shortest follow-up period, 4 years, is 1.73 (95% CI, 1.15–2.04). That estimate is 2.1 times higher than the estimate from Zhang and colleagues but again with higher 95% CI values.

The implications of the findings regarding follow-up period and risk of dementia, AD, and CogImp are discussed in the next section.

## 4. Discussion

As shown in the work of Clarke and colleagues [1], values for biological factors change over time. Therefore, apparent health effects related to those factors are reduced in long-term follow-up prospective studies. Serum 25(OH)D concentrations can change for several reasons.

Vitamin D production from solar UVB exposure decreases with age [30]. A recent experimental study reported that vitamin D production from sun exposure decreases by 13% per decade of life [31].

Serum 25(OH)D concentration is generally inversely correlated with body mass index (BMI; kilograms of mass per square meter of body surface area). For example, in the dementia study from Israel [14], mean BMI was 25 ± 4 kg/m^2^ for 25(OH)D concentrations > 75 nmol/L, increasing to 29 ± 7 kg/m^2^ for 25(OH)D < 25 nmol/L. Thus, if BMI changes, 25(OH)D concentration should also change.

Serum 25(OH)D concentration also is associated with dietary animal product content, especially for fish and meat [32]. If those components of diet change, 25(OH)D will change.

Fortifying food with vitamin D can change 25(OH)D, as it did in Finland, where that approach was approved at the end of 2002 [33]. Measurements of 25(OH)D and dietary assessments of 3650 participants in 1997 at 31 years of age and again in 2012–2013 at 46 years of age determined that fortified foods accounted for most of a 10.6-nmol/L increase in 25(OH)D from 54 ± 19 to 65 ± 19 nmol/L [34].

A 2017 letter to the editor reported changes in daily vitamin D supplementation with 1000 IU or more from data collected in the U.S. National Health and Nutrition Survey [35]. Prevalence for people ≥70 years increased from 1.5% (95% CI, 1.1%–2.0%) in 2005–2006 to 8.6% (95% CI, 5.6%–13.1%) in 2007–2008, and up to 38.5% (95% CI, 31.5%–45.7%) in 2013–2014. The Norwegian study noted that 33% of participants had changes in 25(OH)D concentrations over 10 years [17].

These results have implications for long-duration prospective cohort studies with respect to 25(OH)D concentration. One way is to measure the important factors at least every 4 years. That is the approach taken in Harvard University prospective studies of diet and risk of disease, for example, Bernstein and colleagues [36]. An added advantage of that approach is that the latency period between risk factor and health effect can be determined. In that study, the latency period between dietary meat intake and incidence of colorectal cancer was determined to be about 4–8 years. Another way is to perform analyses for various follow-up periods during the study such as for each 3–5 years without remeasuring the values of the biological factors.

The health benefits of vitamin D status may become apparent much more quickly than for diet in the incidence of adverse health outcomes. For example, a vitamin D randomized controlled trial (RCT) was conducted regarding progression from prediabetes to type 2 diabetes mellitus [37]. The vitamin D dose was 4000 IU/day and the median follow-up time was 2.5 years. When the results were reanalyzed, the HR for diabetes for an increase of 25 nmol/L in intratrial 25(OH)D level was 0.75 (95% CI, 0.68–0.82) in the vitamin D treatment arm and 0.90 (95% CI, 0.80–1.02) in the placebo arm.

The shortest follow-up periods for the prospective cohort studies included in this study were 5.6 years for dementia and AD and 4 years for cognitive impairment. It may be the case that raising serum 25(OH)D concentrations through vitamin D supplementation or UVB irradiance could reduce the risk of developing these brain diseases or slow their progression in much shorter time periods.

The evidence that vitamin D reduces risk of AD was reviewed in 2023 in the *Journal of Alzheimer’s Disease* [38]. Some important mechanisms include reduced risk of insulin resistance (IR) and inflammation. The mechanisms linking brain insulin/insulin-like growth factor resistance include impaired function of glucose transporter 4, changes in insulin receptor function, energy deficit, increased oxidative stress, and hyperglycemia (see Table 1 in Nguyen and colleagues [39]). A 2019 review discussed vitamin D’s role in reducing IR [40]. The mechanisms include maintaining normal levels of reactive oxygen species and ionized calcium, thereby reducing epigenetic changes associated with insulin resistance such as oxidative stress and inflammation.

Therefore, a search was conducted for the effect of vitamin D supplementation regarding health outcomes related to neurodegenerative diseases to ascertain whether supplementation is promising and what time scales are involved. A 3-month study involving elderly people with metabolic disorders showed that supplementation with 2000 IU/day of vitamin D significantly decreased the homeostatic model assessment for insulin and decreased oxidative DNA damage [41]. In addition, supplementation reduced metabolic parameters connected with IR and improved glucose and lipid metabolism.

A 2023 review by Lason and colleagues examined the vitamin D receptor as a potential target for age-related neurodegenerative diseases [42]. The review mentioned a study investigating the effect of vitamin D supplementation involving mild CogImp (MCI) patients [43]. That study included 16 MCI patients, 11 very early AD (VEAD) patients, and 25 healthy control subjects. Patients with 25(OH)D concentrations lower than 75 nmol/L were supplemented with 50,000 IU of vitamin D_3_ once a week for 6 weeks, followed by 1500–2000 IU/day for 18 months. In MCI but not VEAD patients, lymphocyte susceptibility to death improved significantly after 6 months. After 18 months, Montreal Cognitive Assessment scores improved in MCI patients but not in VEAD patients. Because MCI is an important risk factor for AD [44,45], this finding supports the role of higher 25(OH)D concentrations in reducing risk of AD.

In addition, that review [42] included Table 1 with information for eight observational and vitamin D supplementation RCTs regarding late-life cognition, dementia, and AD. Three of those studies reported results of interest for this article. An 18-week RCT compared 4000 versus 400 IU/d vitamin D_3_ effects on visual memory [46]. Participants in the high-dose group increased mean serum 25(OH)D concentration from 67 ± 20 to 131 ± 26 nmol/L, whereas concentration in the low-dose group increased from 61 ± 22 to 86 ± 16 nmol/L. Those in the high-dose group with baseline 25(OH)D concentration < 75 nmol/L increased performance in the Pattern Recognition Memory-Delayed task from 86 (SD 14) to 94 (SD 8) (*p* = 0.005). The change in the low-dose group had *p* = 0.61. No additional significant differences in cognitive function tests were apparent among the other 11 tests for people with 25(OH)D < 75 nmol/L.

A vitamin D supplementation RCT in AD patients conducted in China reported the best results regarding cognitive function [47]. A total of 105 AD patients who received 800 IU/d of vitamin D increased serum 25(OH)D concentrations from 47 ± 7 to 57 ± 4 nmol/L by the end of the year. The 105 participants in the control group decreased 25(OH)D from 49 ± 3 to 47 ± 3 nmol/L. The mean BMI in each group was 25 ± 3 kg/m^2^. People in the vitamin D treatment group had modest increases in full-scale IQ, information, digit span, vocabulary, block design, and picture arrangement, whereas participants in the control group had modest-to-large reductions in all those parameters. The *p*-values for the time and group effects for the vitamin D treatment group compared with the control group were significant to *p* < 0.001 for all but the vocabulary (*p* = 0.15 for time effect) and block design (*p* = 0.02). That RCT showed that vitamin D supplementation could significantly improve cognitive function in AD patients. Thus, that intervention study suggests that vitamin D supplementation can rapidly reduce AD risk factors.

As shown in Figure 1, Figure 2 and Figure 3, RR values increased linearly to the shorter mean follow-up time used for each analysis. However, three studies not included in the regression analysis for CogImp had lower RR than expected from the regression fit to the other six studies. Thus, vitamin D status can affect risk of overall dementia, AD, and CogImp in as little as 5 years. Therefore, any prospective studies of neurodegeneration should measure serum 25(OH)D concentrations at least every 5 years. Harvard has participants in its health studies complete food frequency questionnaires every 4 years [48].

Another measure that should be implemented is to seasonally adjust 25(OH)D concentrations. Many observational studies cited here measured 25(OH)D concentrations at different times of the year and then averaged the values. In the United States, mean adult wintertime serum 25(OH)D concentrations are about 75% of summertime values [49]. In addition, whenever results of meta-analyses of prospective studies are used scientifically or for health policy recommendations, the analyses should be reevaluated with respect to follow-up periods. Also, standardizing 25(OH)D concentration measurements would be helpful since 25(OH)D measured values vary with different assays and instruments. See, for example, Sempos and colleagues (2018) [50].

Low 25(OH)D concentrations have been causally linked to increased risk of AD through Mendelian randomization (MR) studies. MR studies use genetic variants such as alleles of genes involved in the vitamin D pathway to randomize populations. Large-scale vitamin D genome-wide association study (GWAS) datasets are used to determine the relationship between alleles and serum 25(OH)D concentrations [51]. These GWAS data are then used with other large datasets that report health outcomes of interest. With very large datasets, this approach randomizes the effects of other influences on serum 25(OH)D concentration. MR studies are considered capable for causal inference in epidemiological studies [52]. A 2016 MR study found genetically determined serum 25(OH)D concentrations inversely correlated with risk of AD [53]. It included data from an observational study with 17,008 AD cases and 37,154 controls. A 2020 MR study used data from the UK Biobank [51]. It used GWAS data from the International Genomics of Alzheimer’s Project and UK Biobank with individuals aged 60 years and over. Six alleles were used in the analysis. For the International Genomics of Alzheimer’s Project dataset, the OR for AD per one SD increase in 25(OH)D concentation was 0.64 (95% CI, 0.46–0.89). For the UK Biobank dataset, the OR for AD per one SD increase in 25(OH)D concentation was 0.88 (95% CI, 0.73–1.06). The data from the UK Biobank were based on father’s or mother’s history of AD or dementia, an indirect measure of risk. A 2022 nonlinear MR analysis of 25(OH)D concentration and incidence of dementia based on UK Biobank data found for serum 25(OH)D concentration of 10 ng/mL compared to 20 ng/mL and adjusted HR = 1.54 (95% CI, 1.21–1.96) [54]. No RCT has demonstrated that vitamin D supplementation reduces risk of AD. That is probably due to the fact that risk appears to be greatest below serum 25(OH)D concentration = 10 ng/mL, and it is difficult to impossible to enroll enough particapants with such low concentrations in most countries.

Thus, one way to reduce risk of AD is to supplement with vitamin D. A recent review outlined the evidence that supplementing with 2000 IU/day of vitamin D might be an appropriate way for many people to avoid VDD [55]. However, supplementation may not be effective for obese people and may not reduce risk of AD as a result of the higher systemic inflammation from visceral adipose tissue. A meta-analysis of 13 RCTs with 1955 overweight and obese subjects with low 25(OH)D concentrations found that vitamin D supplementation did not influence the inflammatory biomarkers C-reactive protein, tumor necrosis factor-α, and interleuken-6 concentrations [56].

## 5. Conclusions

Vitamin D’s effect on risk of neurological conditions is inversely correlated with mean follow-up period in prospective cohort studies. This effect should be considered in the design and analysis of such studies. As shown in the analysis in this article, using the HR or OR for the shortest follow-up period increases the apparent beneficial effect of high vs. low 25(OH)D concentration on risk of three adverse brain outcomes by about a factor of two compared to analyses without considering follow-up period. Additional studies should also be conducted regarding raising serum 25(OH)D concentrations to reduce risk of brain function decline.

## Data Availability

The original contributions presented in the study are included in the article, further inquiries can be directed to the corresponding author.
